# Discrete ecological gradient in thermokarst ponds in a palsa mire in northern Norway

**DOI:** 10.1038/s41598-025-13046-8

**Published:** 2025-08-04

**Authors:** Ewa Jabłońska, Anders Lyngstad, Izabela Jaszczuk, Jan Kucharzyk, Kristian Hassel, Mateusz Grygoruk, Wiktor Kotowski

**Affiliations:** 1 Faculty of Biology, University of Warsaw, Warsaw, 02-089 Poland; 2 Norwegian Institute for Nature Research, P.O. Box 5685, Torgarden, Trondheim, NO-7485 Norway; 3 Department of Natural History, Norwegian University of Science and Technology, Trondheim, NO-7491 Norway; 4 Centre for Climate Research, Warsaw University of Life Sciences, Warsaw, 02-787 Poland

**Keywords:** Northern peatlands, Vegetation, Finnmark, Šuoššjávri, Hydrochemistry, Moss growth rate, Ecology, Ecology, Environmental sciences

## Abstract

Palsa mires constitute a zonal peatland type in the discontinuous permafrost region of the Northern Hemisphere. They typically consist of permafrost mounds and thermokarst ponds. Global warming has accelerated thawing of permafrost in palsa mounds and an increase in the area of thermokarst ponds in recent decades. Understanding long-term consequences of this process requires in-depth knowledge of the internal diversity of palsa mire vegetation types and their functions. Most studies so-far focused on the palsa mounds. Hereby, we focus on the thermokarst ponds, analysing their vegetation composition and habitat conditions from the top of a palsa plateau down to a fen without current palsa formation close to an adjacent river. We observed a distinct ecological gradient from *Sphagnum*-dominated ponds in the uppermost part of peat plateau to brown moss-dominated fen flarks at the riverside. This reflected well the poor – rich gradient typically recognised in mire vegetation, confirmed by our hydrochemical analyses. However, in contrast to the gradual shifts in species composition along typical mire zonation in temperate regions, palsa microtopography with mounds, rims, strings, and hollows, creates a sequence of mire basins forming a discrete gradient from base-poor to base-rich conditions, allowing different plant species to dominate these distinct locations.

## Introduction

 Palsas are peat-covered mounds, strings, or plateaus with a frozen core of local permafrost^1^. Their formation requires long-lasting mean annual air temperature below − 1°C^1^, thin snow cover to allow deep frost penetration in winter, and low summer precipitation (annual precipitation < 500 mm^2^ to allow dry surface peat to protect the frozen core in summer^3,4^. They do not occur, however, everywhere where climatic conditions are broadly suitable, showing that their formation is determined by numerous local factors as well^3^. Palsas have a circumpolar distribution in the Northern Hemisphere with notable occurrences in Fennoscandia, Siberia, Alaska and Canada^4^.

The extant literature on palsa mire vegetation has predominantly focused on the vegetation of palsa mounds, with comparatively little attention paid to the diversity of the thaw water hollows (thermokarst ponds) within palsas and the fens that surround them. Yet, a comprehensive understanding of this variation is crucial, as it enhances our comprehension of subarctic peatlands and how they are changing due to the thawing of permafrost. This enhanced understanding will facilitate predictions regarding the future transformation of this landscape in response to global warming.

The thawing of permafrost, the degradation of palsas, and the increase in the area of thermokarst ponds has accelerated in recent decades as a consequence of global warming^5^, both in Fennoscandia^6–11^ and in North America^12–14^. In the long term it is suggested that climate-induced permafrost degradation leads to a reduction in the area of thermokarst ponds as they are drained due to increased surface and subsurface hydrological connectivity^15^.

The process of palsa degradation is predicted to accelerate^16^. For Fennoscandia, bioclimatic models suggest that the whole region will be climatically unsuitable for peatland permafrost and palsas by the end of the 21st century^17,18^. As permafrost thaws, greenhouse gases are released, causing high carbon losses^19–22^. Thermokarst-inducing processes play a crucial role in the degradation of permafrost peatlands. Models show that, by the end of the 21st century, thaw-affected soil carbon stocks could be up to twelve-fold of what is projected if thermokarst-inducing processes are ignored^23^. On the other hand, there are observations that thermokarst ponds can be rapidly overgrown with fen or bog vegetation, leading to peat accumulation through the natural successional process of terrestrialisation^12^, suggesting that the thawing of permafrost caused by recent climate change does not necessarily transform palsa mire into a carbon-source ecosystem, as rapid terrestrialisation exacerbates carbon-sink conditions and tends to balance the local carbon budget^12^.

The vegetation that colonises thermokarst ponds depends on where the ice has thawed. If the process occurs along the margin of the raised peat mound, fen vegetation from the surrounding area overgrows the pond^24^. If the collapse occurs within the permafrost body of a palsa, forming a small, isolated reservoir surrounded by permafrost, bog vegetation expands^24^. The macrofossils identified from an isolated thermokarst pond in subarctic Canada show that plant succession followed the pathway: *Calliergon giganteum* and *Sphagnum riparium* when the pond’s depth was at a maximum, *S. riparium* and *C. giganteum* when the pond was partly filled in, and *S. lindbergii* and *S. riparium* when the pond was completely filled in^25^. Another example of the vegetation succession in a thermokarst pond from subarctic Canada shows a similar pattern: aquatic *Sphagnum* species (*S. riparium*,* S. obtusum*,* S. jensenii*, and *S. majus*) as the first colonizers of incipient permafrost collapse scars, followed by the lawn species *S. angustifolium*, and finally replaced by the hummock species *S. fuscum* as the final stage of the terrestrialisation process^26^.

Descriptions of the vegetation of the Fennoscandian thawing palsas are scarce. In fact, the only comprehensive description of the palsa vegetation of northern Norway, including the vegetation in the thermokarst ponds, was published by Vorren from Færdesmyra in eastern Finnmark^27^. In discussing our observations, we refer to his findings. A study from northern Sweden only describes the vegetation of the mound remains and does not show the species overgrowing the ponds that formed after the palsa collapsed^28^.

The main mire vegetation gradients, described from the temperate zone, reflect pH (poor – rich gradient) and fertility^29^. In Scandinavia, however, the poor – rich gradient in vegetation, stretching from rainwater-fed bogs to groundwater-fed fens, encompasses both N- and P-availability (fertility), pH and electrical conductivity^30,31^. The difference in interpretation of the poor – rich gradient is related to the relatively lower nutrient loads in Scandinavia compared to Western and Central Europe^30^. The hummock –lawn – carpet – mud bottom (hummock – mud bottom) gradient, which reflects the distance to the groundwater table, is another major gradient distinguished in Scandinavian mires^30,31^. Palsa mires have a high level of natural disturbance caused by the formation and thawing of permafrost, with steep, or even abrupt, temporal and spatial changes expected along these gradients.

In northern peatlands, bryophytes play a key role in nutrient uptake and cycling^32^. Measuring their growth rate can help to indirectly assess habitat fertility, which is not easily assessed by single in situ measurements of mire water chemistry. Such measurements, however, can easily provide information on pH, so that water chemistry analysis coupled with moss growth rate measurement can provide information on the position of the mire studied on the pH and fertility gradient. The hummock – mud bottom gradient can be described directly from the physiognomy and species composition of the community.

We studied a mire complex including palsa mire in Šuoššjávri, Finnmark county in Norway, where the development and degradation of palsas are relatively well-described^9,33–35^. However, the mire plant communities that cover the thermokarst ponds have not been well studied in this region. We analysed hydrochemical conditions, vegetation composition and moss growth rate in the ponds from the top of the extant palsas down to a fen without present palsa formation closer to the adjacent river. The aim was to improve the understanding of the ecological processes taking place in a thawing palsa mire, emphasizing conditions in thermokarst ponds. We also aimed to describe the differences in vegetation and habitat conditions and ascertain whether they reflect the main ecological gradients of northern peatlands.

## Results

### Vegetation

**Fig. 1:**
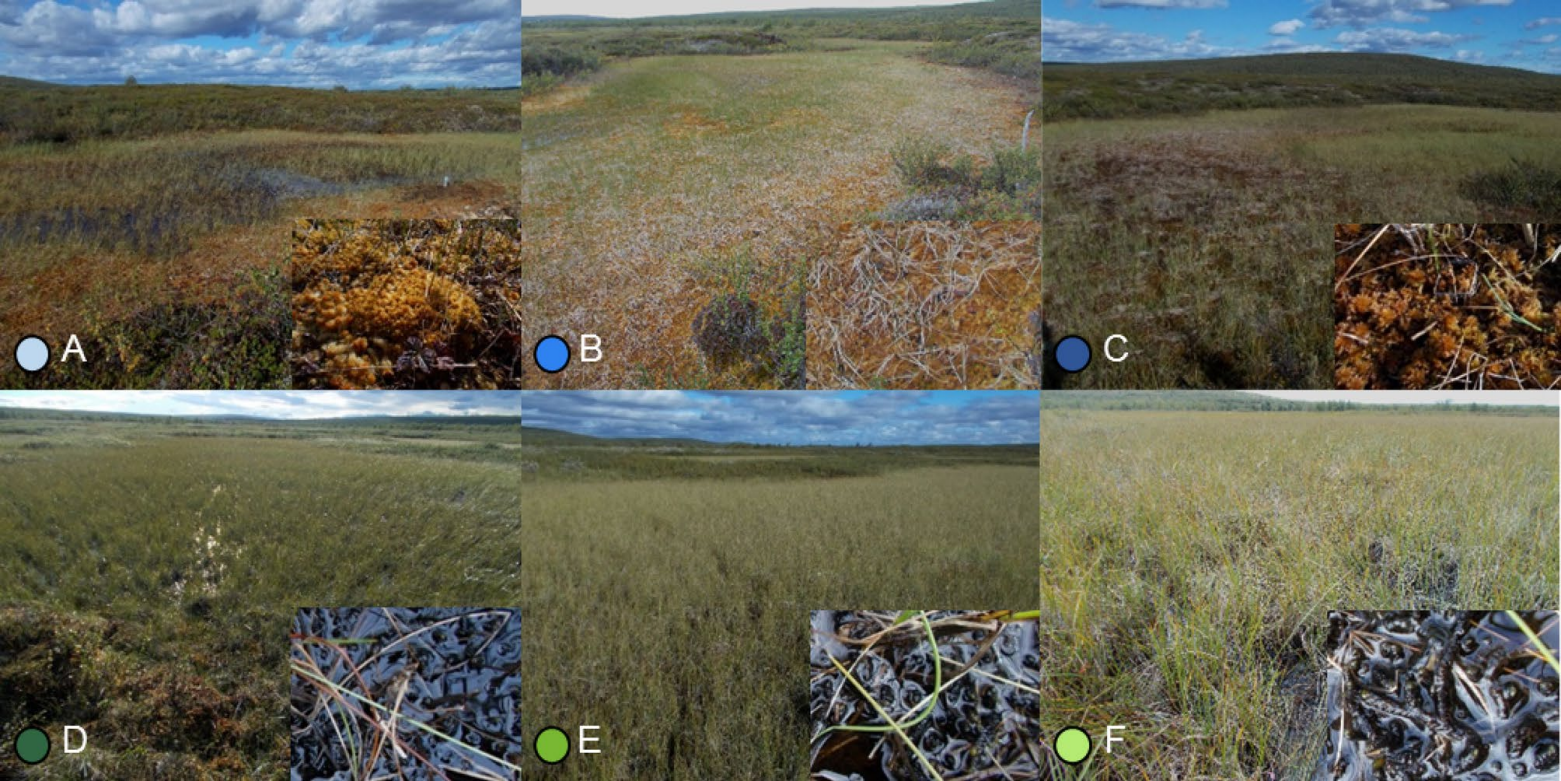
The vegetation of thermokarst ponds (**A**-**F**) in a mire complex at Šuoššjávri, encompassing palsa mire. The ponds represent a gradient from the top of a peat plateau with active palsas (pond A) to an open fen in the vicinity of the Vuottašjohka river (pond F). Colours of points represent different sampling locations (cf. Figures 2, 3, 4 and 5).

The ponds analysed were located in the open mire expanse and had a sparse cover of vascular plants – mostly small sedges (*Cyperaceae*) and cotton grasses (*Eriophorum* spp.). Both the three upper ponds enclosed by palsa peat plateau (A, B and C) dominated by peatmosses (*Sphagnum* spp.), as well as the three lower ponds (flarks) in the fen area without palsa mounds (D, E and F) dominated by submerged brown mosses (Fig. 1), shared a clear resemblance to the suballiance *Caricenion rariflorae* described from the tundra of northwestern Russia^36^ (Table 1). According to the pan-European classifications of bog^37^ and fen vegetation^38^, the vegetation communities of the ponds showed a syntaxonomic gradient from *Sphagnetalia medii* to *Caricetalia davalianae* (Table 1).

**Table 1:**
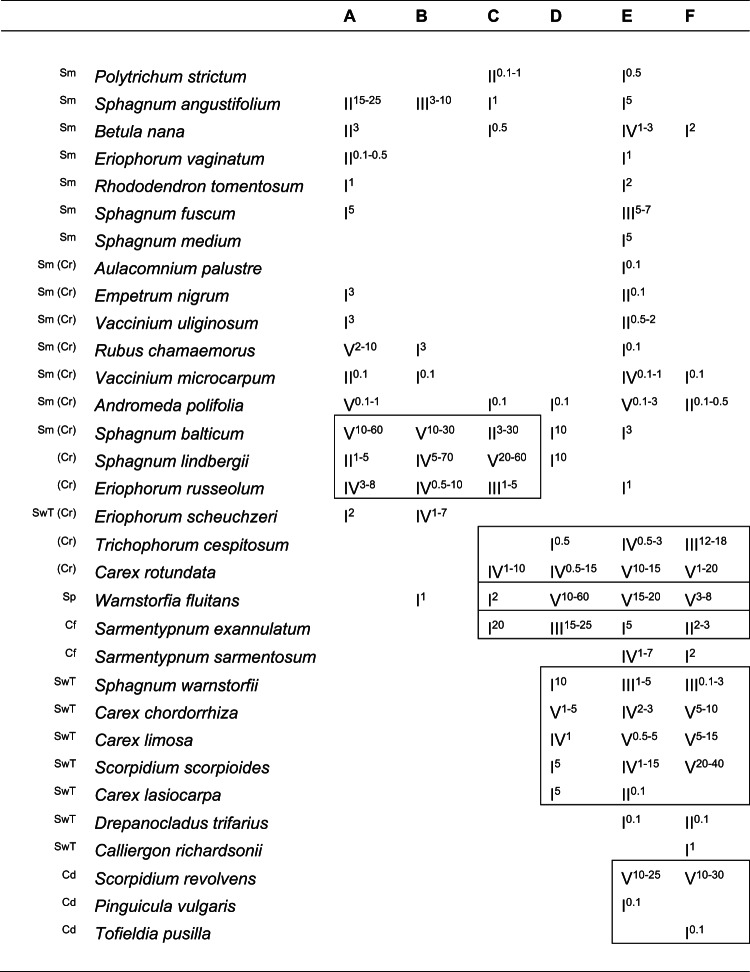
Synoptic table for the vegetation in the ponds A-F. Five relevés were made in each pond. Categorical constancy (I –V) and range of percentage cover (as superscripts) of each species are shown. Only diagnostic species are included (Sm – diagnostic species for the order *Sphagnetalia medii* within the class *Oxycocco-Sphagnetea*^37^. Cd/SwT/Cf/Sp – diagnostic species for the alliances of the *Scheuchzerio palustris-Caricetea fuscae* class within the orders: *Caricetalia davalianae* (Cd), *Sphagno warnstorfii-Tomentypnetalia* (SwT), *Caricetalia fuscae* (Cf), *Scheuchzerietalia palustris* (Sp)^38^. Cr – diagnostic species within the proposed suballiance *Caricenion rariflorae*^36^, *Scheuchzerietalia palustris* order, *Scheuchzerio palustris-Caricetea fuscae* class).

### Habitat conditions

**Fig. 2:**
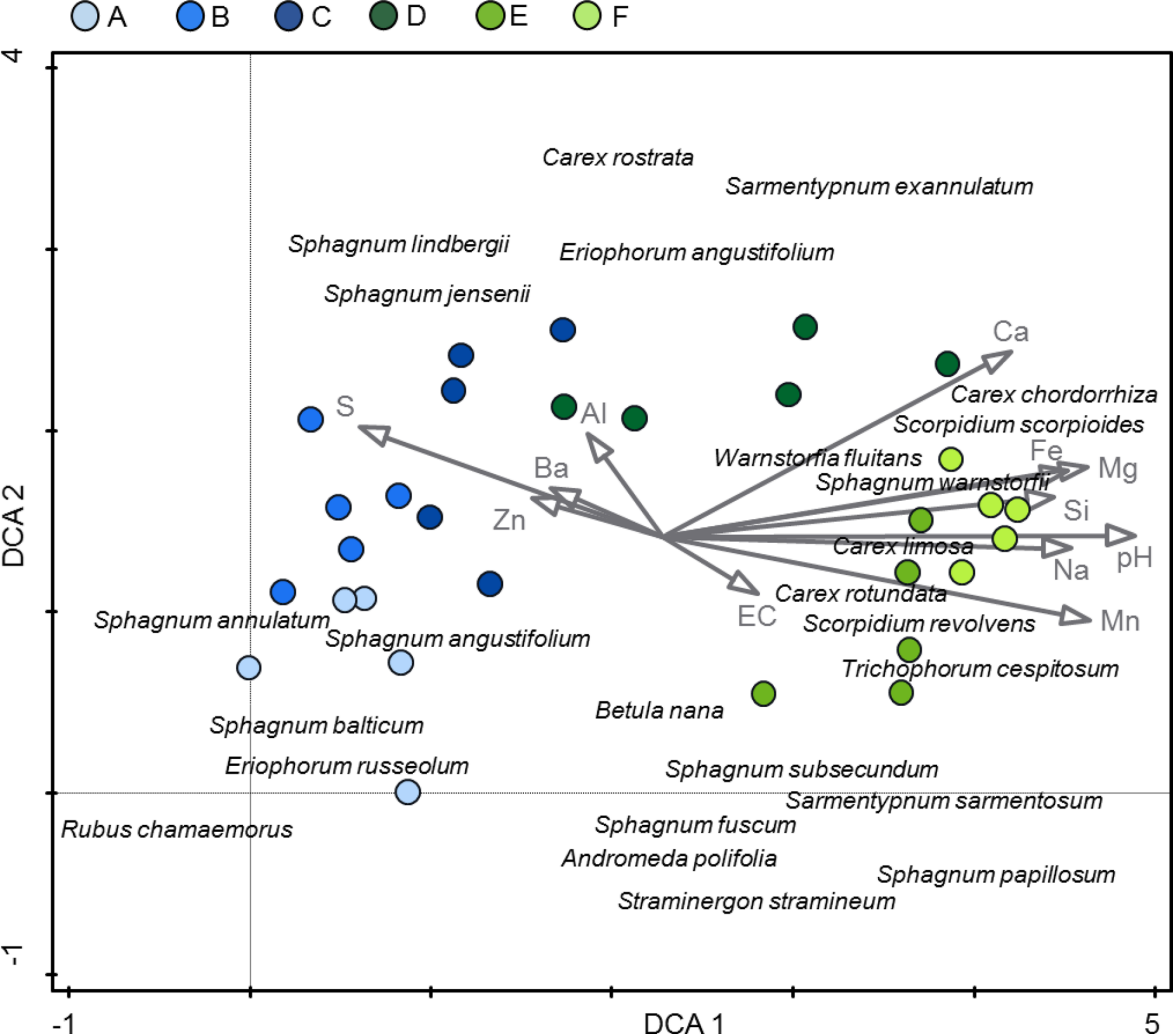
DCA for vegetation plots within the ponds A-F (each of the five plots for a pond shown as a point, 25 species with the best fit to the first DCA axis shown with their names) with water properties as supplementary variables. Eigenvalues: 1st axis – 0.7696, 2nd axis – 0.2396, 3rd axis – 0.1516, 4th axis – 0.0893. Supplementary variables account for 53.4% of the total variation. Colours of points represent different sampling locations (ponds).

**Fig. 3:**
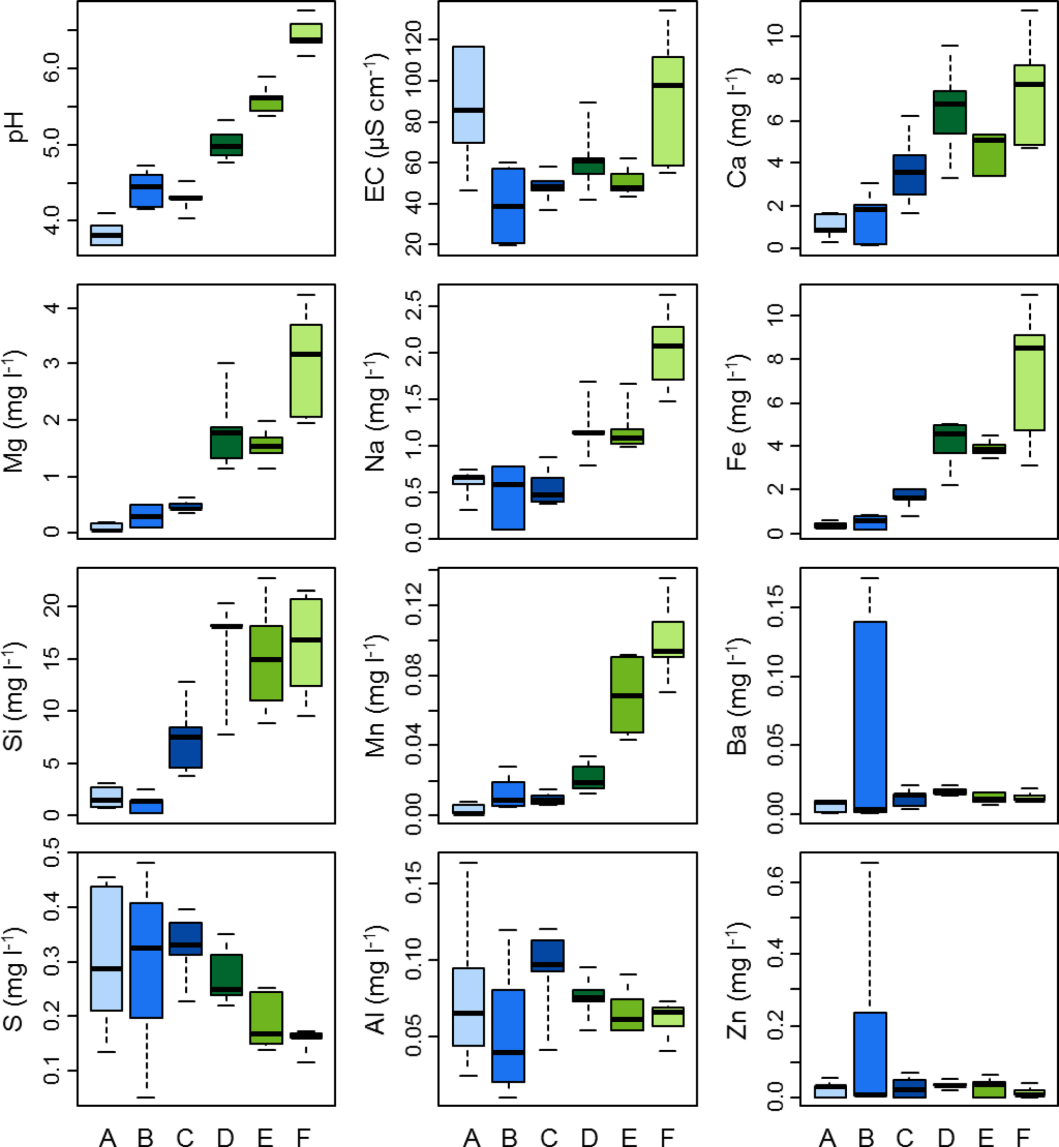
Median, IQR and amplitude of water property values based on 5 samples measured in each of the thermokarst ponds A-F. Colours of boxplots represent different sampling locations (ponds).

The vegetation gradient was coupled with a gradient in habitat conditions (Figs. 2 and 3). The ordination analysis (DCA, Fig. 2) showed that the first axis was related to the poor – rich gradient in vegetation. Rich fen species like *Scorpidium scorpioides* and *Sphagnum warnstorfii* had high scores on the first axis. The *Sphagnum*-dominated ponds A, B and C had comparatively lower pH and lower concentrations of calcium (Ca), magnesium (Mg), sodium (Na), iron (Fe), silicon (Si), and manganese (Mn), but higher concentrations of sulphur (S), barium (Ba), zinc (Zn) and aluminium (Al) in the mire water. The second axis was less clear but appeared to be related to the hummock – mud bottom gradient, with a selection of hummock species scoring low on this axis.

Peat depth was at least 2 m in ponds A and B (our instrument reached down to 2 m), 1.5 m in pond C and 0.5 m in ponds D, E and F. We recorded temperatures above 0 °C throughout the peat layer down to a depth of 2 m during the August 2022 measurement. The temperature of the upper 25 cm peat layer was around 15 °C in all the ponds and decreased with depth to 2 °C at 2 m depth.

### Moss growth rate

**Fig. 4:**
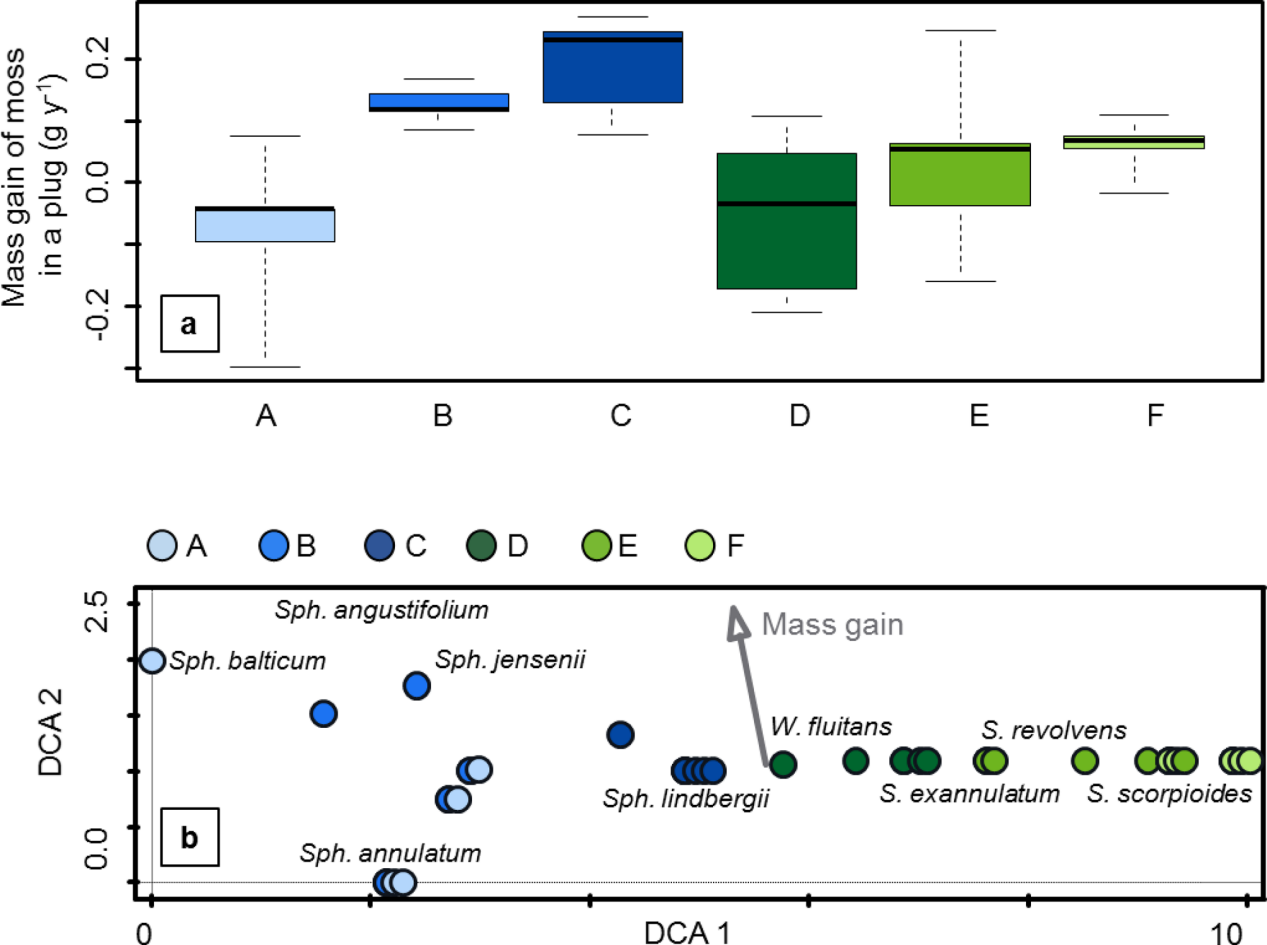
**(a)** Median, IQR and amplitude for moss dry mass gain in the plugs located in the ponds A-F. The area of the plug was 9 cm^2^, so to estimate moss growth in g m^-2^ y^- 1^, the values of moss mass gain should be multiplied by 1111. **(b)** DCA for the species composition in the plugs in the ponds A-F (each of the five plugs for a pond shown as a point, species shown with their names) with mass gain as a supplementary variable. Eigenvalues: 1st axis – 0.9804, 2nd axis – 0.3359, 3rd axis – 0.0813, 4th axis – 0.0327. Supplementary variables account for 5.8% of the total variation. *Sph. – Sphagnum*, *W. fluitans – Warnstorfia fluitans*,* S. exannulatum – Sarmentypnum exannulatum*,* S. revolvens – Scorpidium revolvens*,* S. scorpioides – Scorpidium scorpioides.* Colours of points and boxplots represent different sampling locations (ponds).

Annual net moss growth was highly variable and even sometimes negative (mass loss). Median values, excluding values below zero, were 0.11 g moss dry mass gain in a plug, and 0.07 g when negative values were included (Fig. 4a). The maximal observed mass gain reached 0.27 g dry weight for a plug. Moss mass gain correlated with the second DCA axis, whereas species composition of the patches growing in the plugs formed a gradient along the first DCA axis (Fig. 4b), with no clear relationship between moss species and moss growth rate.

## Discussion

The Šuoššjávri mire complex borders directly on the Vuottašjohka river valley and lake Šuoššjávri. The analysed vegetation gradient extends across the boundary between the permafrost-affected peat plateau with active palsa mounds in the central area of the mire complex and the fen area closer to the river without current permafrost. This covers a distinct gradient from the recent thermokarst ponds A, B and C to the fen flarks D, E and F which may represent older thermokarst ponds. The peat plateau boundary in this area is possibly prone to spatial shifts due to its proximity to the river. Water level is among the most important factors influencing the dynamics of palsas. Palsas located on a floodplain, close to a riverbed, are considered more affected by thawing than those located further away from the river^13^. Palsas are often absent at the margins of large rivers with temporary flooding, and the proposed mechanism causing this is that the presence of water in the topmost layers of palsas during the thawing season causes their degradation^8^. In the case of Šuoššjávri, the hydrological connection of ponds D-F with the groundwater probably explains their difference from ponds A-C, which are isolated from the groundwater by the frozen peat. According to the flood risk map^39^, flooding of the entire Šuoššjávri mire complex by the Vuottašjohka river is possible. However, based on the current wetland vegetation of the area, flooding seems to be a defining ecological factor for the riparian forest along the river and not for the area with the ponds we studied. Direct contact of the palsa base with groundwater has also been described as a reason for extensive palsa thawing in Færdesmyra^27^.

Thermokarst lakes can rapidly terrestrialise due to lateral drainage, which promotes encroachment of mire vegetation^40^. Circular depressions created by thawing are mainly overgrown with bog vegetation^41^, but the composition and cover of the vegetation varies considerably with the age of the drainage^42^. In Alaskan tundra, *Carex aquatilis* and *Eriophorum angustifolium* dominates recently drained thermokarst ponds where shallow water is still present, shifting to communities of *Carex stans*, accompanied by e.g. *Sphagnum* spp. in terrestrialised wet ponds^42^. We did not analyse the age of thermokarst pond formation and terrestrialisation. Nevertheless, our observations suggest that the *Sphagnum*-dominated pond A is the youngest in our gradient – the pond A is the closest to the centre of the peat plateau and it is the most quaking of the palsa plateau ponds A-C (Fig. 1.). In Færdesmyra the newly forming floating mat over a terrestrialising palsa thaw pond was dominated by *Sphagnum annulatum*,* S. lindbergii*,* S. angustifolium and Eriophorum russeolum*^27^, showing a clear resemblance to our pond A. However, pond A is dominated by *S. balticum*, which in Færdesmyra is a species attributed to carpet and lawn communities representing a later stage in the successional trajectory of ponds^27^. Based on this, we suggest that pond A is in the second stage of terrestrialisation already. Other carpet-lawn species in Færdesmyra thaw ponds were *Warnstorfia fluitans* and *Carex rotundata*. Soak vegetation with *C. rotundata* was described from Færdesmyra as receiving thaw water from many surrounding palsas but also upstreaming water from deeper layers^27^. We observed *C. rotundata* in the groundwater-influenced ponds C-F.

The vegetation diversity in the studied gradient is likely largely influenced by the source of the water feeding the patch – with rainwater dominating at the top of the palsa (pond A) and the influence of groundwater increasing closer to the river. This corresponds well with the poor – rich gradient in mire vegetation. We also found a clear gradient of pH as well as Ca, Mg, Na, Fe, Si and Mn in water taken from the surface layer of the mire along the A-F transect, with all the values increasing towards the river (Figs. 2 and 3). The above elements indicate that the water is of groundwater origin. This also coincides with lower peat depth in the ponds D-F compared to the ponds A-C on the palsa plateau, suggesting that a shorter distance to groundwater is reflected in both hydrochemical conditions and vegetation.

Pond F (closest to the river) has up to 10 mg l⁻¹ Ca, 4 mg l⁻¹ Mg, 2.5 mg l⁻¹ Na, 10 mg l⁻¹ Fe (Fig. 4). It is covered by rich fen vegetation with *Caricetalia davalianae* species (Table 1). The concentration of Ca is markedly lower in comparison to the e.g. Central European lowland fens with *Caricetalia davalianae* vegetation, where it reaches about 40 mg l^−1^^43^ and far lower in comparison to the Carpathian fens with *Caricetalia davalianae* vegetation, where Ca content reaches even up to 190 mg l^-1^^1,44^. The concentrations of Mg and Na are here also somewhat lower in comparison to the Central European and Carpathian *Caricetalia davalianae* rich fens. Fe content is slightly higher in our pond F than in the compared Central European *Caricetalia davalianae* rich fens^43^ but lower than the values reported from Carpathian rich fens^44^. On the other hand, Si concentration in pond F is twofold higher than the values from Carpathian sites^44^. The difference in elemental content compared to Central Europe, especially the much lower Ca content, is due to the geological structure of the area. The bedrock of the Šuoššjávri is a large plate of granitic gneiss and granite^45^, i.e. rocks poor in calcium carbonate. However, probably due to the low degree of water buffering, the pH is relatively high at low Ca levels, which leads to the formation of moss and small sedge communities of *Caricetalia davalianae*, typical of rich fens.

The vegetation gradient from the low-lying fen area to the top of the palsa plateau is made up of plant communities that reflect a gradient of decreasing pH, ending in the acidic pond A with its bog community. According to the pan-European classification of mire vegetation^37,38^, this is a clear gradient from *Caricetalia davalianae* to *Sphagnetalia medii* (Table 1). The predominantly bog-like vegetation of the ponds A-C still has, however, some locally distinctive indicators of minerotrophy: *Eriophorum russeolum*,* Sphagnum annulatum* and *S. angustifolium*^27^. The thermokarst ponds within the bog plain can act as “fen windows” – the subsurface water may be forced upwards there^27^. Nevertheless, the observed ecological gradient in palsa ponds still clearly corresponds to the poor – rich gradient^29,31^. However, in contrast to the gradual shifts in species composition typically encountered along typical mire zonation, palsa mires create a sequence of mire basins forming a discrete gradient from base-poor to base-rich conditions, allowing different plant species to dominate these distinct locations. This physical separation between mire ‘zones’ may suggest that interspecific competition is of less importance here as a factor structuring vegetation patterns as compared to continuous zonation gradients. In addition, the relatively slow growth of the bryophyte species measured in this study may further limit the importance of interspecific competition.

Our moss growth measurements should be interpreted with caution, as the number of replicates available in this study was probably insufficient to fully capture and understand the causes of growth rate variability. Despite these limitations, our results provide interesting preliminary insights. Although there were clear vegetation gradients, moss growth rates were similar across the ponds, with high variability even within a single pond. We assumed that growth rates in *Sphagnum*-dominated ponds would be slightly higher than in brown moss-dominated ponds^46–49^, resulting in the manifestation of a gradient in moss growth rates between ponds, but our results do not support this assumption. The maximum mass gain observed in our study was 0.27 g dry mass per plug, corresponding to approximately 300 g m^-^² y^-^¹. The median growth rates were significantly lower: 0.11 g dry mass per plug (120 g m^-^² y^-^¹) excluding negative values and 0.07 g dry weight per plug (79 g m^-^² y^-^¹) including negative values. These results are consistent with previously reported values for fen brown mosses, which typically range from 55 to 229 g m^-2^ y^-1^, with a maximum of 659 g m^-2^ y^-1^, and in the case of the mosses from *Polytrichaceae* family up to 800 g m^-2^ y^-1^^47–50^. *Sphagnum* mosses have a global mean dry mass production of 259 (± 206, SD) g m^-2^ y^-1^, with a minimum of 8 g m^-2^ y^-1^ and a maximum of 1450 g m^-2^ y^−1^^48^. As expected, brown moss growth measured in palsa mires is lower than growth in temperate mires^50^.

The climatically induced, ongoing loss of palsas is likely to intensify^4,17,18^, and by the end of this century, new formation of palsas will not be possible throughout large areas of the current palsa mire region. In the long run, this will likely reduce the local hydrochemical and ecological diversity of mire complexes as the compartmentalization among thermokarst ponds weakens. On a landscape scale there is, however, reason to believe that the carpet vegetation now seen in thermokarst ponds will be maintained, because the mire massif types (e.g. islet mixed mire or string fen^2^) likely to replace palsa mires also support mire features like hollows, pools and flarks.

The large scale thawing of palsas represents a significant disturbance of vegetation communities and the peat itself, causing mineralization of peat and release of macronutrients. While the details for palsa mires remain obscure, we can hypothesize that increased nutrient availability will enhance plant growth, and certain graminoids are particularly well adapted to increase their biomass, while bryophytes are outcompeted^51^.

Apart from the impact on the vegetation itself, this will also add to the impact on GHG-balance of these peatland areas. Graminoids with aerenchyma are known for their ability to efficiently vent methane from deep peat layers, circumventing the methanotrophs associated with e.g. *Sphagnum* mosses^52^. Other likely impacts on GHG-balance are increased CO_2_-sequestration due to increased temperature, longer growth periods and increased nutrient availability, but also increased methane production due to wetter conditions. The overall impact of the role of thermokarst ponds on GHG-balance is a topic well suited for further studies.

The thawing of palsa mires does not necessarily entail a large net loss of carbon from the peat. Increased peat decomposition may well be counterbalanced by increased accumulation, implying that the function as a carbon sink is maintained^53^. In the long run, palsa mires may even be replaced by peatland types with higher peat production potential, e.g. plane bog or other ombrogenous mire types. It is therefore worth noting that despite the likely demise of palsa mires as a mire massif type throughout much of its present distribution, the protection of these peatlands from land use change is no less important. To better understand peat accumulation potential of different mire vegetation types of the Arctic, it is worth continuing the in-situ studies of the growth rates of bryophytes and vascular plants and complementing them with studies of decomposition rates, that were not included in our study.

Summing up, the vegetation of thermokarst ponds in a thawing palsa mire at Šuoššjávri represented a gradient from the rich fen of the *Caricetalia davalianae* to the bog of the *Sphagnetalia medii*. This corresponds with the bog – poor fen – rich fen gradient. The hydrochemical properties followed a similar pattern to the diversity of vegetation. Moss growth was rather low, and the growth rates were similar across the ponds irrespective of bryophyte species and the pH values. The ordination analyses revealed that some of the vegetation variation was also likely related to the hummock – mud bottom gradient. Interestingly, the mire features of a palsa mire, with palsa mounds, rims, strings, and hollows, isolated the thermokarst ponds to such an extent that their vegetation appeared more as discrete zones than a continuous gradient.

## Methods

### Study site

**Fig. 5:**
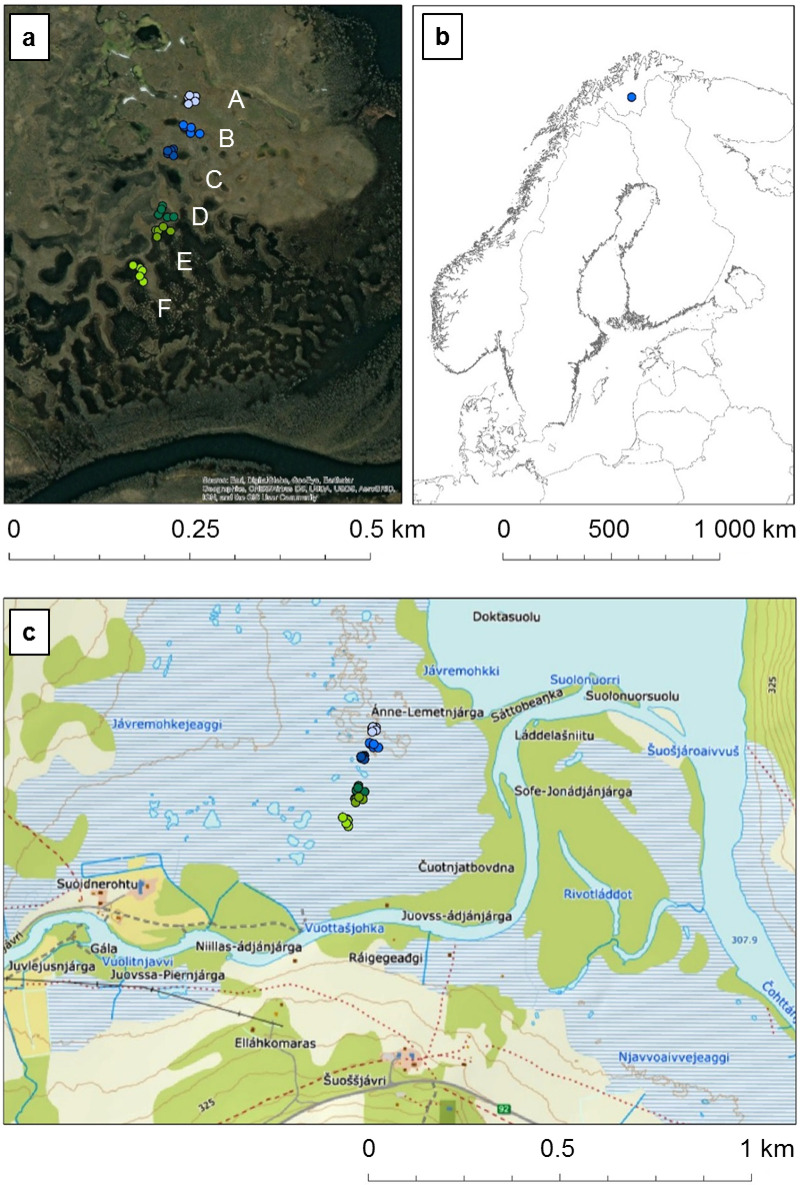
**(a)** Six thermokarst ponds analysed (**A**-**F**) with the location of the five sampling plots for each pond shown by dots in a different colour for each pond. In the background, there is an aerial photograph of the study site^66^. **(b)** Study site location shown on Northern European border outline, © 2025 Michael Bauer Research GmbH. **(c)** Sampling plots shown on a topographical map of the Šuoššjávri area, a screenshot of which was copied from ©norgeskart.no^67^, licensed under CC BY 4.0. Colours of points represent different sampling locations (ponds). The maps were compiled using ArcGIS Desktop 10.5, version 10.5.0.6491, ©1999–2016 Esri Inc.

We investigated a palsa mire located near the Šuoššjávri settlement in northern Norway, on the Finnmarksvidda plateau (Fig. 5). We selected six adjacent thermokarst ponds (A-F, Fig. 5) along a gradient starting from the top of the palsa mounds in the central palsa plateau to the fen areas currently without permafrost closer to the Vuottašjohka river. Finnmarksvidda, located in the interior of the Finnmark county has a subarctic climate, corresponding to the northern boreal and alpine vegetation zones^54^. The study site is underlain by permafrost^55^. The 1991–2020 average annual climate normals for Šuoššjávri^56^ are: precipitation – 577 mm, daily maximum temperature – 2.6 °C, daily minimum temperature – −6.3 °C, daily mean temperature – −1.6 °C. The mean annual air temperature for Šuoššjávri increased by more than 1 °C for the period 1995–2014 compared with the period 1967–1980, and mean annual precipitation increased by around 60 mm for the same period^33^. A snow cover of more than 25 cm was present in Finnmarksvidda for an average of 100 to 200 days per year during the period 1991-2020^57^. The mean maximum snow depth in Šuoššjávri from 1967 to 2014 was around 60 cm^33^.

The Finnmarksvidda climate conditions are changing significantly due to global warming. The average annual temperature for the county is estimated to increase by approximately 5.5 °C until 2100^58^. The largest temperature increase is calculated for winter, with a significant reduction in the amount of snow and the number of days with snow. The growing season is forecasted to increase by 1–3 months. Areas with permafrost will be significantly reduced over the course of this century^58^.

The study site is located within a large palsa mire massif including an elevated peat plateau on permafrost. Peatland inception here is dated to 9787 cal. years BP, while permafrost aggradation is dated to 118 cal. years BP^34^. The Šuoššjávri palsa plateau extends over approximately 23 ha^59^. The plateau degrades more slowly than the surrounding smaller palsa mounds. 19% of the original area of this plateau in 1956/1959 had degraded by 2012, whereas in the same period, 59% of the area of smaller palsa mounds in this mire complex had collapsed^33^. For example, the small thermokarst ponds 170 m and 200 m west of our pond F were formerly palsa mounds that collapsed between 2003 and 2014, with no permafrost observed in 2014^33^. The pond 390 m north of our pond A (located on the palsa plateau) also degraded between 2003 and 2014, but in August 2014 some permafrost was still evident in the palsa remnants, with an active layer of 45 cm^33^. Thermokarst degradation is concentrated along the margins of the peat plateau, representing 77% of the observed subsidence, while most of the inner plateau surface exhibits no detectable subsidence^35^. Our ponds A, B and C were collapsed exclaves within the palsa plateau as early as 1956, whereas our ponds D, E and F are located in a fen outside of the current boundary of the main palsa plateau^9,33^.

### Moss growth rate

To measure moss mass gain (g y^-1^), we used the plug method^60^. In August 2022, we planted a total of 30 plugs, i.e. five replicates from each pond, containing undisturbed patches of species dominant in each pond. Accurate identification of individual stems was only possible under the microscope after the experiment, so the plugs may have contained a mixture of several species. We took 9 cm^2^ samples (the horizontal cross-sectional area of the plug) from the moss layer, cut each one to the length of the plug (4.5 cm), weighed their wet mass, and placed them in the plugs, which were then returned to the moss layer. After one year, in August 2023, we collected the samples, dried them at 75 °C for 24 h and weighed them again. We calculated the mass gain by subtracting the initial dry mass from the final dry mass. The initial dry mass was calculated from the initial wet mass using a regression equation prepared on the basis of the results of measurements of an additional ten moss samples taken from each pond in 2022^60^.

### Vegetation characteristics

In August 2023, we took 30 vegetation relevés of a 1 m^2^ area, the centre of which was the location of a plug used in the moss growth experiment. A percentage scale was used to measure plant cover. We named mosses after^61^ and vascular plants after^62^.

### Habitat conditions

Water samples were taken from 0 to 9 cm below the soil surface in each of the 30 sampling plots. Rhizosphere’s MacroRhizon device was used with a membrane pore size of 0.12–0.18 μm. Elemental concentrations were determined by inductively coupled plasma optical emission spectroscopy (ICP-OES). Measurements were carried out by a Perkin-Elmer OPTIMA 8300 spectrometer. Peat depth and soil/groundwater temperatures were measured using a manual, portable probe equipped with a temperature sensor (WTW^®^ LF 320; ±0.05 °C resolution)^63^ in each of the six ponds.

### Data analysis

Vegetation data, habitat characteristics and data on moss growth rate were analysed using boxplots in R^64^ and Detrended correspondence analysis (DCA) in Canoco5^65^. The length of gradient in both DCA analyses exceeded 4 SD, which confirmed the validity of using the unimodal ordination model. Species data were log-transformed.

## Data Availability

The datasets generated during and analysed during the current study are available from the corresponding author on reasonable request.
